# Dimeric IgG complexes from IVIg are incapable of inducing *in vitro* neutrophil degranulation or complement activation

**DOI:** 10.1371/journal.pone.0195729

**Published:** 2018-04-10

**Authors:** Iwan Kustiawan, Ninotska I. L. Derksen, Theresa Guhr, Simone Kruithof, Wim Jiskoot, Gestur Vidarsson, Theo Rispens

**Affiliations:** 1 Department of Immunopathology, Sanquin Blood Supply and Landsteiner laboratory Amsterdam Medical Centre, University of Amsterdam, CX, Amsterdam, the Netherlands; 2 Division of Drug Delivery Technology, Cluster BioTherapeutics, Leiden Acedemic Centre of Drug Research (LACDR), Leiden, the Netherlands; 3 Department of Immune heamatology, Sanquin Blood Supply and Landsteiner laboratory Amsterdam Medical Centre, University of Amsterdam, CX, Amsterdam, the Netherlands; Institut National de la Santeet de la Recherche Medicale (INSERM), FRANCE

## Abstract

**Purpose:**

Intravenous immunoglobulin (IVIg) products contain various amounts of dimeric IgG complexes. Current insights into the possible biological activities of these dimers remain controversial, and both immunemodulating and immune-activating effects have been reported. Here, we analyzed the putative immune-activating effects of dimers isolated from IVIg.

**Methods:**

Dimers isolated from IVIg were purified by high-performance size-exclusion chromatography (HP-SEC) and tested for the ability to induce neutrophil degranulation in vitro.

**Results:**

Dimers isolated from IVIg were found to be incapable of inducing in vitro neutrophil degranulation or complement activation, even at concentrations exceeding those expected to be reached upon administration in patients. These results depend on the removal of artefactual activation by using 0.1 micron filtration and the use of poloxamer to prevent adsorption of IgG onto the solid phase.

**Conclusions:**

The data suggest dimeric IgG found in IVIg may bind to Fc-receptors without causing activation.

## Introduction

IVIg is a pool of IgG derived from thousands of healthy blood donations. IVIg has been used for more than 30 years in the clinic for immune deficiency and autoimmune diseases treatment[1]^,^[2]^,^[3]. The administration of IVIg, however, is not without side effects. The incidence of unwanted effects was 12–23% for patients receiving IVIg[4]. The adverse reactions are usually mild and include headache, chills, fever, flushing, dizziness, malaise, and chest tightness. It is suggested that the effects are associated with the increase of pro-inflammatory cytokines and vasodilators triggered by components in IVIg, such as dimers or aggregates during adminstration[5],[6]. The side effects tend to disappear if the infusion rate is decelerated or by using steroidal anti-inflammatory drugs[7].

In early years, when IgG was fractionated only by cold ethanol fractionation for intramuscular administration, large amounts of aggregated IgG (up to 30%) could be detected in the final product[8]. The aggregation might be a result from hydrophobic contact or elevated temperature during production[9]. This aggregated IgG might induce cross-linking of IgG-Fc receptors (FcγR) on effector cells and complement cascade activation[10]^,^[11],[12] leading to the release of pro-inflammatory factors. In the 1990s, additional production steps for intravenous application were introduced to remove aggregates by lowering temperature, pH and ethanol concentration, adding proteolytic enzymes such as pepsin and re-fractionating using ion-exchange chromatography. Since then, the presence of IgG aggregates (larger than dimers) in IVIg is regulated up to 3% as a requirement for product release[13].

Although the production process of IVIg products has been improved, a substantial amount of dimer remains present (4–11%) in the final product[14]. These dimers are assumed to be mainly the result of Fab-Fab interactions between idiotypic and anti-idiotypic antibodies.[15]^,^[16]. The concentration of dimeric IgG in IVIg is positively related with the number of donors contributing to the IgG pool, which is thought to raise the number of idiotype-anti-idiotype combinations[17,18]. Other factors influencing dimerization include concentration, pH and temperature.

The role of dimers in IVIg during treatment remains elusive. Both beneficial, immunomodulatory effects as well as side effects have been ascribed to the presence of dimers in IVIg. With respect to the immunomodulatory effects, both *in vitro* studies in mice and *in vitro* studies using human macrophages have suggested an immune-suppressive effect being exerted by dimeric IgG[19]^,^[20],[21]. Also, a positive correlation was observed between the dimeric IgG concentration in CIDP patient’s blood post IVIG treatment and clinical improvement.[22] On the other hand, another study failed to demonstrate enhanced immunosuppressive activity from dimers in a mouse ITP model.[23] Also, neutrophils isolated from CIVD patients treated with IVIg have a similar CD16, CD11b and Siglec 9 receptor expression levels and respond similarly to bacterial stimuli, indicating a lack of phenotypical alterations in circulating neutrophils upon IVIg treatment[24]

In contrast to potential beneficial effects, other studies suggest that side effects of IVIg treatment may in part be the result of neutrophil activation via dimeric IgG. Some studies using a rat model suggest that dimeric IgG induces a pro-inflammatory effect during IVIg administration. Dimeric IgG is thought to be responsible for hypotension induced by either complement-dependent macrophage activation or by FcγR dependent neutrophil activation[25]^,^[26]^,^[27]. Furthermore, multiple in-vitro studies have indicated that dimeric IgG from fractioned IVIg increases FcγR mediated oxidation burst and calcium influx of human neutrophils[28]^,^[29]^,^[30]. However, as the monomeric IgG fraction also activated neutrophils, albeit to a lower degree in these studies, this begs the question whether the reported activation by the dimer may be over-represented or even artefactual. Interestingly, one in vitro study reports induction of oxidative burst of neutrophils exposed to ‘low’ (up to 5 mg/mL) concentrations of IVIg as opposed to high (> 10 mg/mL) concentrations of IVIg[31]

We recently found that commonly used methods for *in vitro* neutrophil stimulation assays is actually caused by IgG adhering to the material used to carry out the experiments. This causes FcγR-crosslinking and activation, explaining how monomeric IgG can cause neutrophil stimulation in these assays[32]. Furthermore, a method was developed to overcome this limitation. In the present study we assessed the effects of dimeric IgG fractions isolated from IVIg on neutrophils *in vitro*. In contrast to previous work, we found that dimeric IgG does not activate neutrophils even at very high concentrations.

## Materials and methods

### Antibodies

The IVIg product used in this study was Multigam (CAF-DCF, Belgium). Mabs used were human CD32a (Novoprotein, NJ) and CD16a (Sino biologicals Inc., China), rabbit anti-human elastase (Sanquin, the Netherlands), anti-C4-1 monoclonal antibody (mAb) (Sanquin, the Netherlands).

### Reagents

Poloxamer 407 (BASF, the Netherlands), PBS (phosphate buffered saline, 10 mM phosphate, pH 7.4, 140 mM NaCl, Fresenius Kabi, the Netherlands), IMDM (Iscove's modified Dulbecco's medium, Lonza, Belgium), human elastase (EPC, Missouri, USA), cytochalasin B (Sigma, Germany), N-formyl-Met-Leu-Phe (fMLP; Sigma, Germany). PTG (PBS supplemented with 0.1% Tween 20 and 0.2% gelatin A), TMB (3,3′,5,5′ tetramethylbenzamidine, Uptima INTERCHIM, France), streptavidin horseradish peroxidase (HRP) (Amersham Life Science, UK), 96-well maxisorb plate (Nunc, Denmark), 96-well polysorb plate (Nunc, Denmark). Biacore buffer HBS-PE (GE Healthcare, the Netherlands). Ampex red (Molecular Probes/Thermo Fisher Scientific, US), HRP (Sigma, Germany).

### Isolation of neutrophils

Peripheral blood from anonymous, healthy volunteers was obtained with informed, written consent in accordance with Dutch regulations. This study was approved by the Sanquin Ethical Advisory Board in accordance with the Declaration of Helsinki. Neutrophils were isolated from with an isotonic Percoll gradient method. Neutrophils were purified from erythrocytes by lysing erythrocytes with lysis buffer (0.15 M NH_4_Cl, 0.01 M KHCO_3_, and 0.1 mM EDTA) (Verhoeven et al., 1988)[33].

### Fractionation of IVIg’s components

IVIg’s components were fractionated with high-performance size-exclusion chromatography (HP-SEC) on an AKTA system (GE Healthcare, Sweden) using a Hiload 16/600 Superdex 200 column and PBS as elution buffer. The dimeric fraction and monomeric fraction were pooled and concentrated using Amicon filter centrifuge tubes (Millipore, US) to a concentration of 30 mg/ml. The fractions were dialyzed against IMDM and filtered through a 0.1-μm syringe filter before being used for further experiments.

### Neutrophil activation

Isolated neutrophils of male healthy volunteers were diluted with IMDM media supplemented with 100 IU/ml (w/v) penicillin, 100μg/ml streptomycin and 5% fetal calf serum (Biowhittaker, USA) to a concentration of 4 X 10^5^ cells/ml. 25 μL of cell suspension was incubated with different concentrations of 50 μL IVIg fractions (dimeric or monomeric IgG) supplemented with 0.2% poloxamer 407 and IMDM was added to total volume 100 μL. For the experiments with primed neutrophils, the cells were incubated with cyto B 5 μg/ml for 10 minutes or recombinant human TNF (Active Bioscience, Hamburg, Germany) 25 ng/mL for 30 minutes before incubation with IVIg fractions. As negative control, neutrophils were incubated with IMDM media supplemented with 0.2% poloxamer 407; as positive control, 5 μg/ml and 1 nM of cytochalasin B (Sigma, Germany) and fMLP (f-Formyl Met-Leu-Phe; Sigma, Germany), respectively, were added. The cells and stimulant were incubated at 37°C for 1 hour followed by centrifugation. The supernatant was taken and directly stored at -20°C before elastase ELISA analysis.

### Elastase ELISA

Rabbit anti-human elastase (Sanquin, the Netherlands) was diluted in PBS to 1.5 μg/ml. 100 μL of diluted antibody was added into the wells of a maxisorp 96-well plate and stored overnight in the fridge (2–8°C). The 96-well plate was washed 5 times with PBS containing 0.05% Tween 20 (PBS-T) and 100 μL sample (100-fold diluted in PTG buffer) or human elastase standard (10 ng/ml serially 2-fold diluted in 11 different concentrations) was added. The sample was incubated at room temperature for 1 hour under gentle shaking and washed 5 times with PBS-T before incubation with 100 μL biotinylated rabbit anti-human elastase antibody (1 μg/ml) for another hour. The plate was washed 5 times with PBS-T and further incubated with 100 μL streptavidin HRP (1:1000 in PTG) for 30 minutes. After washing with PBS-T 5 times, 2-fold diluted TMB substrate was added and the reaction was stopped after 13 minutes with 2 N sulfuric acid. The absorbance was read by using a Titertek Multiscan (Flow Laboratories, Mc Lean, VA) at 450/550 nm. The concentration of elastase was calculated by extrapolating the absorbance of sample to the standard elastase curve’s absorbance.

### Oxidation burst assay

50 μL of 10^6^ neutrophils in HEPES medium was incubated at 37°C for 30 minutes in black 96-well plates, followed by adding 100 μL reaction solution containing 1 U/ml HRP and 0.05 mM Amplex red freshly prepared in HEPES. Incubation was continued for 5 minutes and 50 μL of samples (IVIg or IVIg fractions in several concentration ranges) or positive controls (0.04 mM fMLP and 0.04 mM PAF in HEPES medium) were added. Samples were incubated in a plate reader for 1 hour while oxidation burst was being measured at Ex 535 nm, Em 590 nm and a reading interval 30 s.

### Complement activation

The ability of IVIg fractions to activate complement was assessed by combining samples diluted in veronal buffer supplemented with 0.02% Tween-20 with a serum pool and incubating the mixtures for 1 hour at 37°C. Heat-aggregated IgG (pooled human immunoglobulin 10 mg/mL in PBS, incubated at 63°C for one hour) was used as positive control. Afterwards, production of C4b was quantified as a measure for complement activation, as described before[34]. Briefly, anti-C4-1 mAb was coated onto an ELISA plate. Serial dilutions of the sample were incubated in the wells for 1 h at 4°C. After washing, biotinylated polyclonal anti-C4 antibodies were used for detection. A titration curve of normal serum that had been incubated at 37°C for 7 days in the presence of 0.02% (w/v) NaN_3_ (NSA) was used as a standard. The level of activated C4 was expressed as a percentage of the amount of C4b/c present in NSA or as nmol/l (NSA contains 2500 nmol/l C4b/c).

### Surface plasmon resonance

The IVIg fraction and His-tagged human recombinant receptors (CD32a and CD16b) were dialyzed against Biacore buffer and diluted to several concentrations prior to surface plasmon resonance (SPR) analysis. SPR measurements were performed with a Biacore 3000 system (Biacore AB, the Netherlands) at 25°C. Anti-histidine antibody (GE Healthcare, the Netherlands), 25 μg/ml in sodium acetate buffer, pH 4.5 (GE Healthcare, the Netherlands), was coupled covalently to a CM5 Chip (GE Healthcare, the Netherlands) following activation with EDC (1-ethyl-3-(3 dimethylaminopropyl)-carbodiimide, Sigma, Germany) 0.4 M in water and NHS (N-hydroxysuccinimide, Sigma, Germany) 0.1 M. To measure the binding of polyclonal IgG to FcγRs, human recombinant polyHisTag FcγRIIa or human recombinant polyHisTag FcγRIIIb was diluted in HBS EP buffer (10 mM HEPES, pH 7.4, containing 3.4 mM EDTA, 0.15 mM NaCl, and 0.005% v/v Tween 20; GE Healthcare, the Netherlands) and injected, followed by the injection of IgG (0.16 to 5 μM) diluted in HBS EP buffer. A blank run, consisting of injection of receptor followed by injection of buffer, was used as reference run; data was corrected by subtracting results from this run. The data was processed with BIAevaluation 3.2 RC1 software. Fitting of a Langmuir binding model to Rmax values vs IgG concentrations was done with GraphPadPrism.

## Results

### Fractionation of IVIg and characterization of IVIg fractions

**Fig 1:**
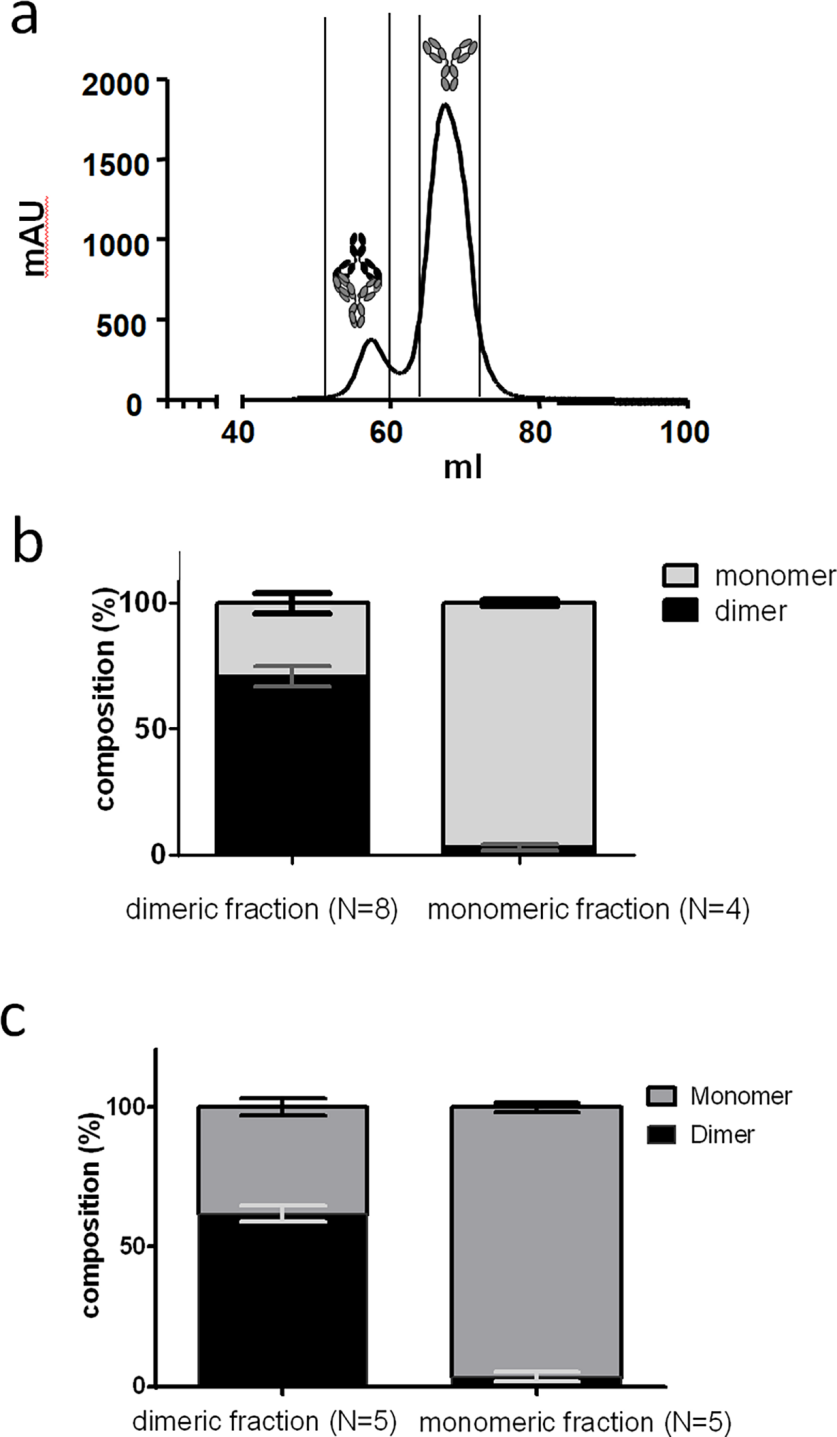
Fractionation of IVIg. Fresh IVIg was injected onto a HP-SEC column and elution profiles were recorded at 215 nm. Dimeric IgG was separated from monomeric IgG; a representative chromatogram is shown in (a). Each fraction was collected separately and further analyzed with an analytical HP-SEC column. Dimeric IgG partially dissociated to monomer, whereas only small amounts of monomeric IgG associated to form dimer (b). The fractions were incubated at 37°C for 1 hour to resemble condition in neutrophil activation experiment. The composition each fraction was analyzed with an analytical HP-SEC column (c). Error bars indicate mean ± SD.

The IVIg product used in this study (Multigam) consisted of 5.6±1% dimeric IgG and 94.4±1% monomeric IgG (Fig 1A). Fractions representing monomeric or dimeric IgG were pooled as indicated in Fig 1A and concentrated. Immediately after fractionation and concentration, about 30% of the dimeric fraction had dissociated into monomeric IgG. At the same time ca. 3% of the monomeric fraction seemed to have associated to form dimeric IgG (Fig 1B). We also analyzed the monomer and dimer content of both fractions after incubation for 1 hour at 37°C, diluted to 2 mg/mL in IMDM, representing the conditions during incubation with neutrophils (Fig 1C). About 60% of IgG in the dimeric fraction remained dimeric, while the composition of the monomeric fraction remained similar.

### Dimeric and monomeric IgG do not activate neutrophils in vitro

**Fig 2:**
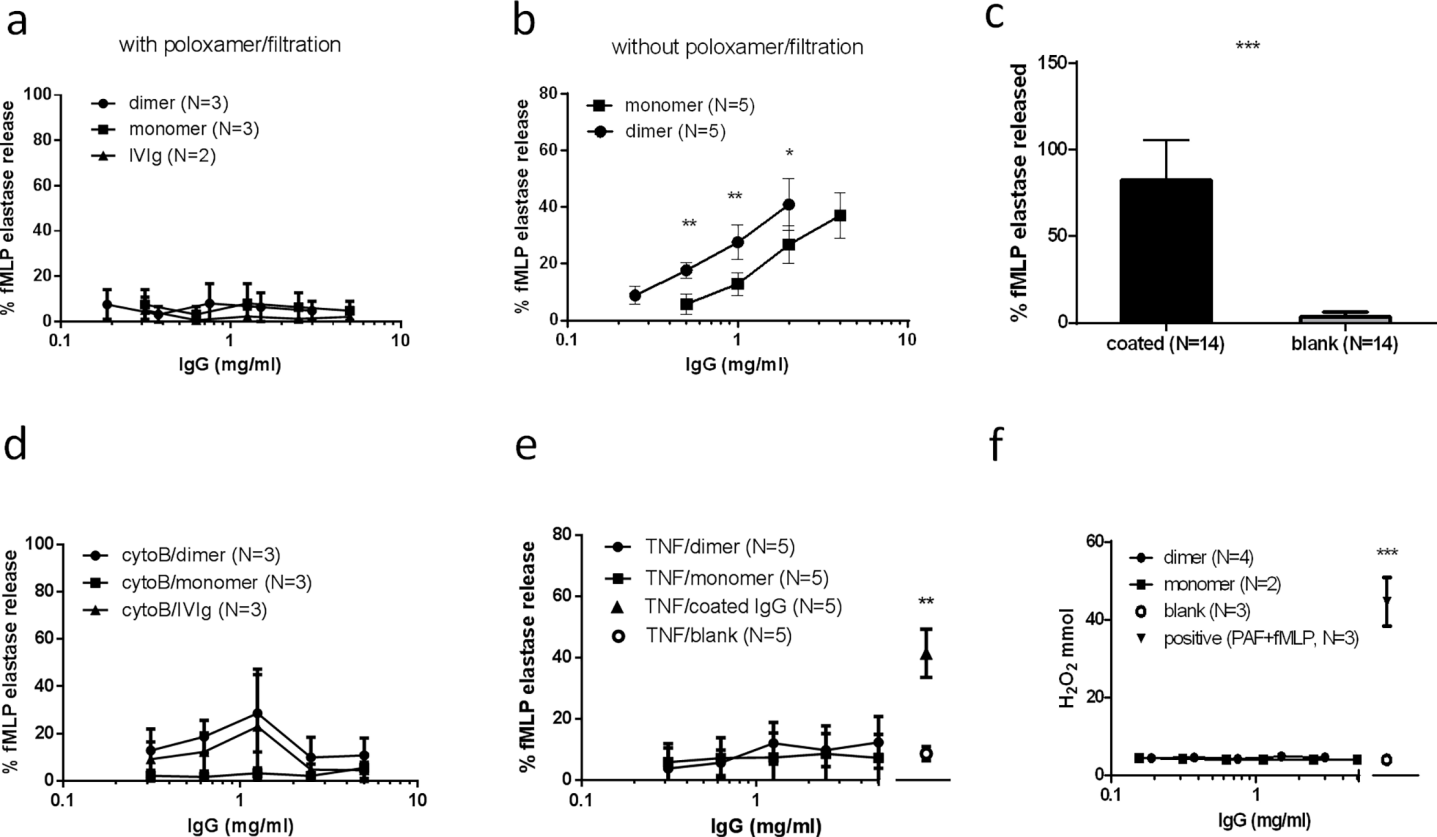
Neutrophil activation by IVIg and fractions thereof. Neutrophil activation, measured as elastase release of resting neutrophils, induced by filtered IVIg fractions at concentrations between 0.3 and 5 mg/mL in the presence of poloxamer (a), or using a previously established protocol without filtration and poloxamer (b). As a positive control, neutrophils were also stimulated by coated IgG, which leads to more than 80% degranulation at concentrations as low as 0.5 mg/mL (c). Activation of primed neutrophils using cytochalacin B (d) or TNF (e) by filtered IVIg fractions in the presence of poloxamer. Similarly, oxidation burst was evaluated in the presence of filtered IVIg fractions in the presence of poloxamer (f). One-way ANOVA (a, d, e, f) or Student’s t test (b, c); * p < 0.05, ** p < 0.01, *** p < 0.001.

To study the direct effects of dimeric IgG on neutrophils (i.e., dimers in solution), we filtered samples from monomeric and dimeric fractions of IVIg (at concentrations between 0.3 and 5 mg/mL) with an 0.1 micron filter, and used poloxamer to prevent neutrophil activation by surface-coated IgG, as described before[35]. None of the fractions caused degranulation of neutrophils (Fig 2A). By contrast, using a protocol where filtration and the use of poloxamer was omitted, degranulation was observed for both dimeric as well as monomeric fractions (Fig 2B). When using poloxamer, but omitting the filtration step, we never observed activation with IVIg, but did sometimes observe activation with the dimeric fraction, and to a lesser extent with the monomeric fraction. IgG coated onto a solid surface also resulted in activation of neutrophils (Fig 2C). Degranulation by IVIg fractions was also assessed for neutrophils that were primed with either cytochalasin B or TNF, to facilitate degranulation. However, even under these conditions, neither a clear neutrophil degranulation (Fig 2D and 2E) nor induction of an oxidative burst (Fig 2F) was observed.

### Complement activation

**Fig 3:**
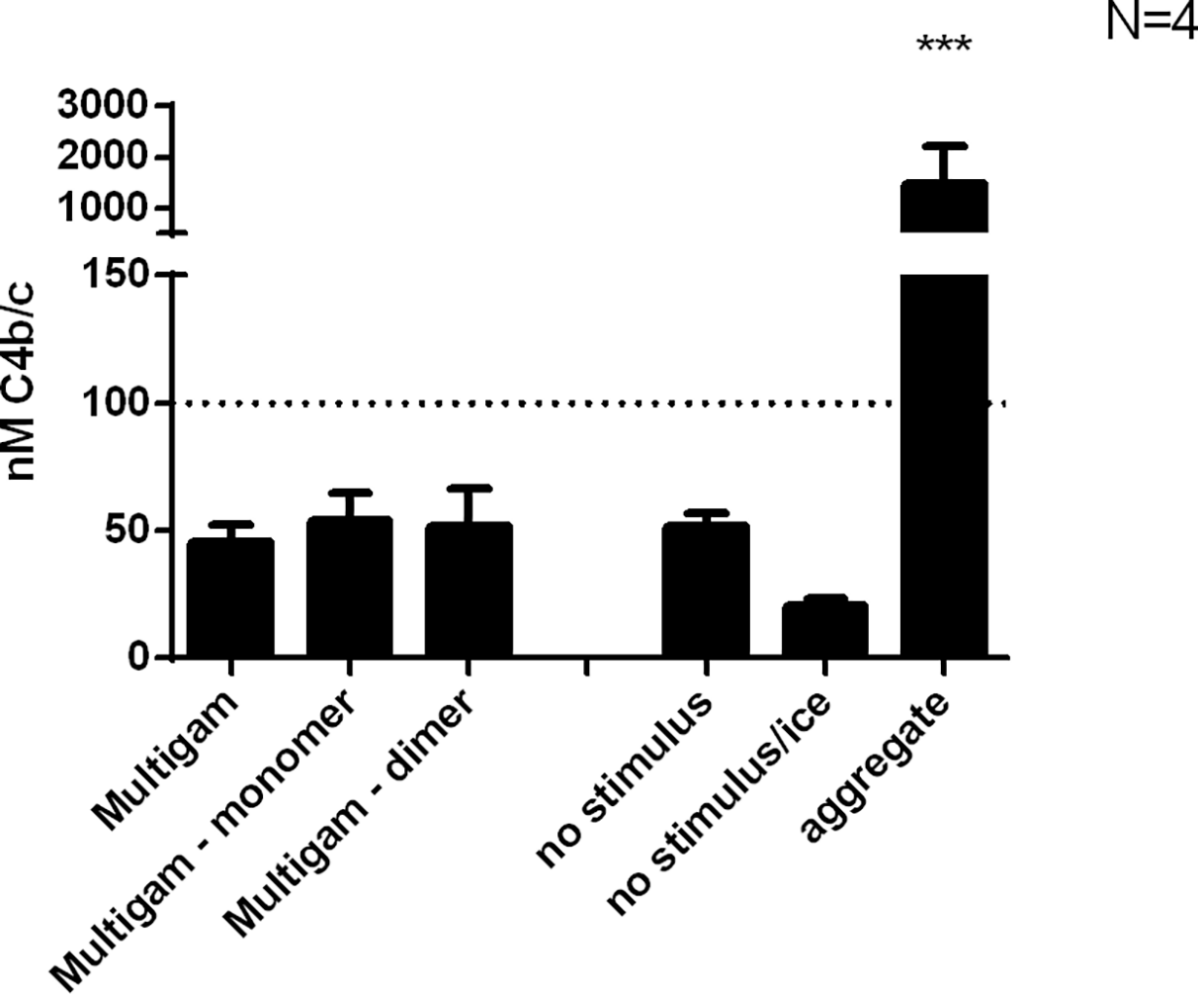
Complement activation by IVIg fractions. Detection of fluid-phase complement activation by measuring C4b/c in serum incubated at 37°C together with IVIg, dimeric, monomeric fraction and heated IgG (aggregates), or no stimulus, incubated at 37°C or on ice. One-way ANOVA; *** p < 0.001.

Besides crosslinking of FcγRs induced by immune complexes, antibodies can activate complement. Higher order IgG multimers or aggregates have increased affinity for C1q, with an optimum for hexameric IgG. Minimal complement activation was seen as C4b/c from the monomer or dimer fraction or the unfractionated IVIg, except after heat aggregation (Fig 3).

### Apparent binding affinity to FcγRIIa and FcγRIIIb of dimer fraction is higher than that of monomer fraction

**Fig 4:**
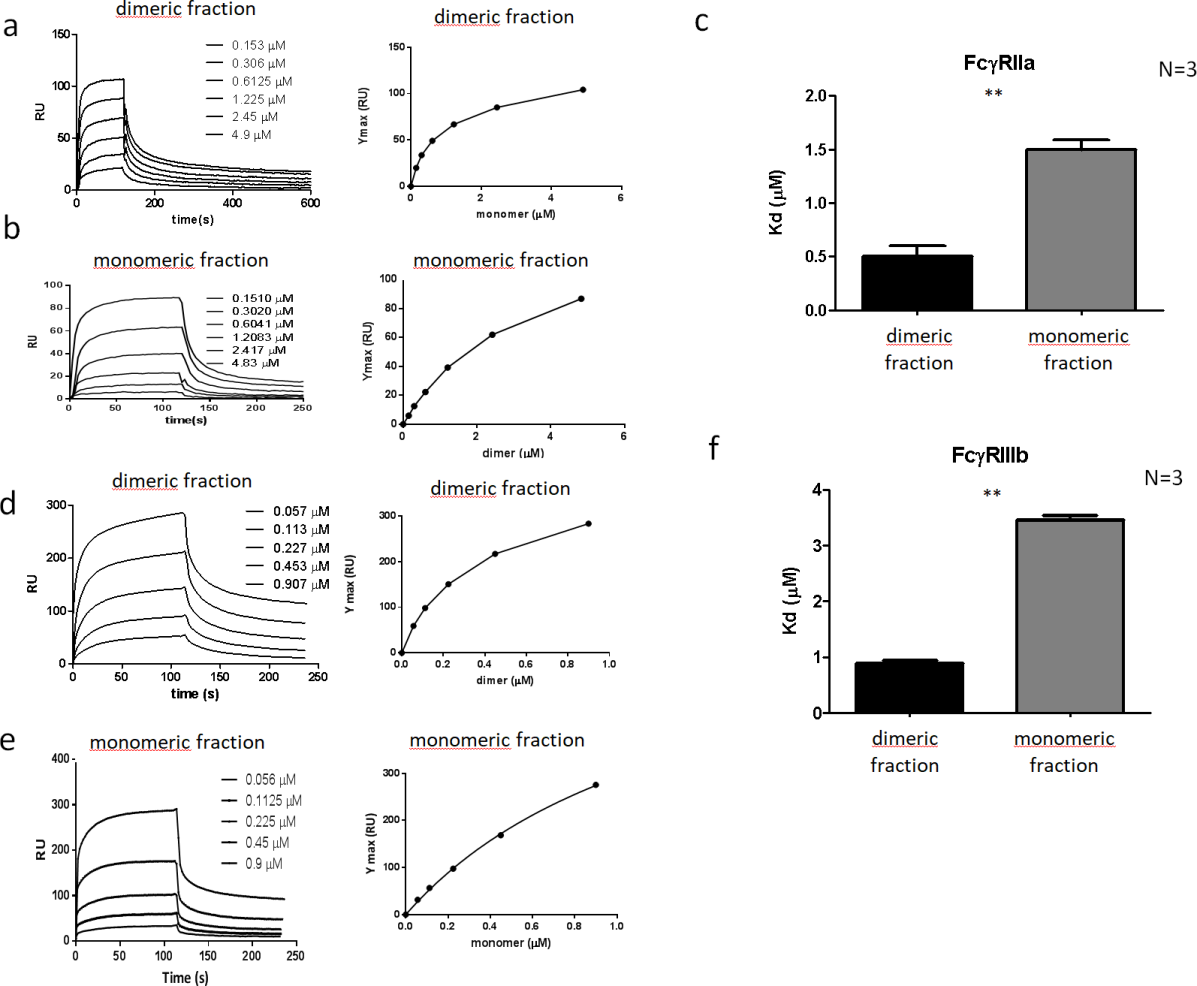
Binding to FcγR by IVIg fractions. Sensograms of the binding of dimeric fraction (a,d) and monomeric fraction (b,e) to FcγRIIIb (a-b) or FcγRIIa (d-e) coupled to a biosensor chip through its C-terminal His-tag. Steady state apparent affinities calculated from the sensogram data were obtained from fitting a Langmuir binding model as shown in the inset panels, to calculate the apparent Kd values to FcγRIIIb (c) or FcγRIIa (f). One-way ANOVA; ** p < 0.01.

We also analyzed the binding of monomeric and dimeric IgG fraction to C-terminally His-tagged FcγRIIa and FcγRIIIb, mimicking their natural orientation in the neutrophil membrane. The FcγRs were first allowed to bind to anti-His-coupled biosensor chips, and their binding to the IgG fractions was assessed by SPR. The SPR data indicate that dimeric IgG had a higher apparent affinity to both FcγRIIa and FcγRIIIb compared to monomeric IgG (Fig 4).

## Discussion

Despite a long history of IVIg in the treatment of immune deficient and autoimmune disorders, the role of dimeric IgG in IVIg remains elusive. Immune complexes, including dimers, are generally perceived as unwanted due to their potential to induce inflammation. However, the present study suggests that dimers derived from IVIg are poor activators of both resting and primed neutrophils as well as complement, even at or exceeding the high concentrations associated with IVIg treatment.

Study of the effects of IVIg and its fractions in in vitro assays is complicated, because very high concentrations of IgG are required to resemble the in vivo situation upon administration of IVIg, i.e., a range of ca. 1–10 mg/mL. These very high concentrations easily result in variable degrees of IgG absorbing to solid surfaces like microtiter plates, even in the presence of abundant amounts of other proteins (albumin). The absorbed IgG can induce activation of cells such as neutrophils, the most abundant white blood cell present in human blood[35].

Previously we found that poloxamer can eliminate neutrophil activation at these high IgG concentrations by preventing IgG adsorption in test tubes[36]^,^[35]. Polysorbate is also effective but will lead to cell lysis. Here, we found that in addition to the use of poloxamer, 0.1 micron filtration of fractionated dimers and monomers was necessary to completely prevent neutrophil activation. This filtration step did not significantly affect the overall IgG concentration or dimer content of the fractions. One possible explanation for the activation that was sometimes observed with the unfiltered fractionated material is that small amounts of aggregates were formed during the process. The amounts of aggregates likely differ between successive fractionations, resulting in different degrees of activation. Preliminary analyses using nanoparticle tracking analysis (NTA) revealed the presence of particles in the range of 150–300 nm predominantly in the dimer fraction, which indeed could be effectively removed to undetectable levels by filtration through a 0.1-μm filter without significantly affecting the dimer and monomer composition of the fractions. Nevertheless, this does not prove that these aggregates are causing the activation.

Our results are in line with studies showing a stronger induction of neutrophil degranulation by insoluble immune complexes compared to (smaller) soluble complexes[37]^,^[38]^,^[39]. An IgG dimer is the smallest possible immune complex (containing two IgG molecules) and therefore the least effective in cross-linking multiple Fc receptor molecules. Likewise, recruiting the C1 complex probably requires more than two IgG molecules, or preferentially hexamers spatially organized through Fc-Fc interactions, allowing for maximal C1q interaction.

Perhaps not surprisingly, our experiments revealed that binding affinity/avidity of dimeric IgG to FcγR is higher than that of monomeric IgG. Although the exact receptor density on the biosensor surface may not be representative of densities on the actual cellular surface, we did do our best to simulate the actual orientation, by attaching the receptors to the SPR biosensor through a C-terminal His-tag. However, in contrast to a cell membrane, there is no possibility for lateral movement of the receptor molecules. Therefore, these binding studies cannot reveal to which extent dimers from IVIg may bind more efficiently as compared to monomeric IgG. Nevertheless, it is likely that dimers also bind more avidly to Fc receptors on effector cells[40]. Given their poor ability to activate, as shown in this study, this suggests that dimers might be more potent inhibitors of Fc receptor-mediated effector mechanisms, thereby contributing to the anti-inflammatory effects of IVIg. This is in line with a recent study demonstrating recombinant Fc trimers to be effective inhibitors of Fc receptor-mediated monocyte or neutrophil activation[41].

Indeed, Teeling et al. reported the prevention of platelet clearance by a dimeric IVIg fraction in rats[20]. However, to obtain dimeric IgG, reconstituted freeze-dried IVIg was stored at 4°C until the concentration of dimeric IgG gradually increased. This method probably results in formation of larger aggregates as well as dimers, and larger IgG complexes might have (disproportionally) contributed to the observed effects. In addition to this, chemically stabilized dimerized IVIg was found to be more effective in preventing idiopathic thrombocytopenic purpura (ITP) in a mouse model[21]. On the other hand, another study reported no clear correlation between the dimer content of human immunoglobulin products and their effectiveness in ITP mouse models[23]. One factor that complicates these studies is the dynamic and reversible nature of the dimerization process in these products[18], with temperature, concentration, pH, and ionic strength all affecting the amounts of dimers. This implies that following injection into mice, the amounts of dimers may change, which might explain the apparent lack of correlation. Furthermore, IVIg may contain autoantibodies to neutrophil proteins including siglec-9 that are highly enriched in dimeric fractions, and have been suggested to also contribute to an anti-inflammatory effect of IVIg [42]

The experiments in this study were performed using isolated neutrophils, which potentially influences the results. However, neutrophils isolated by density gradient centrifugation has been reported to become more prone to degranulation,[43] which would only strengthen our conclusion that dimers are incapable of triggering degranulation.

In conclusion, dimeric IgG from IVIg is a poor trigger of neutrophil degranulation and complement activation. The results from this study do not support the notion that dimeric IgG in IVIg is a major cause of adverse reactions.
